# Coconut oil and medium-chain fatty acids attenuate high-fat diet-induced obesity in mice through increased thermogenesis by activating brown adipose tissue

**DOI:** 10.3389/fnut.2022.896021

**Published:** 2022-10-28

**Authors:** Yunxiao Gao, Yiwen Liu, Xue Han, Fang Zhou, Jielong Guo, Weidong Huang, Jicheng Zhan, Yilin You

**Affiliations:** ^1^Beijing Key Laboratory of Viticulture and Enology, College of Food Science and Nutritional Engineering, China Agricultural University, Beijing, China; ^2^College of Health Solutions, Arizona State University, Phoenix, AZ, United States

**Keywords:** obesity, coconut oil, medium-chain fatty acids, brown adipose tissue, thermogenesis

## Abstract

Coconut oil (CO) and its main ingredients, medium-chain fatty acids (MCFA), present many benefits. Whether MCFA and CO play an equally valuable role in anti-obesity remains unclear. This study compared the anti-obesity effects of CO and MCFA [octanoic acid (C8:0) and decanoic acid (C10:0)] to gain insight into the underlying mechanism. Male C57BL/6J mice were fed either a low-fat diet (LFD) or high-fat diet (100% HFD) replaced with 2.5% MCFA (97.5% HFD + 2.5% MCFA) or 5% CO (95% HFD + 5% CO) for 17 weeks. CO and MCFA ameliorated the HFD-induced abnormal body and adipose depot weights, insulin sensitivity, and energy expenditure (EE), which was associated with brown adipose tissue (BAT) thermogenesis. Furthermore, CO enhanced the expression of thermogenesis markers in BAT, which was consistent with increased BAT activity. CO showed a better effect than MCFA in activating BAT to increase thermogenesis and energy metabolism to combat obesity, which may be attributed to the cooperation of MCFA and other substances in CO. This work provides evidence for the anti-obesity effects of CO, which could be a better alternative to lard in daily diet, rather than pure MCFA.

## Introduction

Obesity has become a serious global disease accompanied by hypertension and type 2 diabetes mellitus (T2DM) (1). Recent research has shown that obese people are also less resistant to severe COVID-19 (2). Obesity first appeared as a disease in the 1990s due to the consumption of more processed foods high in fat and sugar, especially fructose (3). Furthermore, obesity is exacerbated by reduced physical activity and increased sedentary behavior (4). Although an excess intake of fat results in overweight or obesity, fat plays a crucial role in nutrition by contributing to texture, flavor, nutritional value, and caloric density (5).

As the exploration of the nutritional effects of different types of fatty acids increases, coconut oil (CO) has been widely accepted as a healthy option due to its unique fatty acid composition (6). However, it is precisely because of its high content of saturated fatty acids (92%), the role of CO in cardiovascular disease is still controversial (7–9). CO contains high concentrations of medium-chain fatty acids (MCFA), such as typical octanoic acid (C8:0) and decanoic acid (C10:0), as well as vitamins and polyphenols (10). This unique composition of CO provides it with distinctive digestion, absorption, and metabolic characteristics (11). MCFA are widely concerned ingredients in CO for their energy supply characteristics. After intestinal absorption, MCFA enter the portal vein mainly in a non-esterified form and can be used as a rapid energy source in muscles, the liver, and heart tissues (12). Lauric acid (C12:0), which has distinctive metabolic characteristics, including rapid energy conversion, that are not found in long chain fatty acids (LCFA, C14–C18), is still ambiguously classified since some studies define MCFA as C6-C10 only (12–14). The MCFA used in this work do not contain lauric acid. While LCFA impair insulin sensitivity and lipid metabolism, MCFA seem to protect against obesity and associated metabolic derangements (11). MCFA also stimulate the thermogenesis of brown adipose tissue (BAT) and triggers marked elevation of thermogenesis features at the hepatic level to promote weight loss, although the specific mechanism is not fully understood (15). It is worth noting that most of the conclusions regarding MCFA use CO as a raw material source in the study. Research shows that MCFA and CO cannot be considered equal even though CO exhibits relatively high MCFA concentrations (16). Furthermore, it is necessary to provide a more rigorous assessment of the impact of a diet which uses MCFA alone vs. another which uses CO.

Current ways to lose weight include exercise, drugs, surgery, and other strategies, which are either challenging to persist with or have several side effects. One emerging strategy involves stimulating metabolically active tissues, such as brown and beige adipose tissues, to release excess energy. The presence of uncoupling protein 1 (UCP1) on the inner membranes of the BAT mitochondria is essential since it allows H^+^ to enter the exterior mitochondrial membrane without passing through adenosine triphosphate (ATP) synthase, dissipating the potential energy of the protons used to synthesize ATP in the form of heat energy (17). Moreover, mature white adipocytes can be transformed into brown fat-like adipocytes when exposed to specific stimuli, such as cold conditions and β3-adrenergicreceptor (β3-AR) agonists (18). These characteristics render BAT stimulation a promising anti-obesity strategy. However, cold stimulation is challenging to achieve, while the increase in blood pressure caused by β3-AR agonists is particularly harmful to obese patients (19, 20). Therefore, it is essential to find ingredients that can safely and effectively stimulate BAT activity, preferably based on diet.

Previous studies have explored the respective amelioration effect of CO and MCFA on obesity (10, 21). However, whether MCFA can play the same anti-obesity role as CO remains unclear, and the specific mechanism involved warrants further exploration. Therefore, the composition of CO is analyzed. Furthermore, the role of CO and its main ingredient, MCFA, consist of octanoic acid (C8:0) and decanoic acid (C10:0) in this work, in resisting obesity and related complications is determined, comparing the effect of MCFA with CO. Since activating BAT to increase energy consumption is a potential mechanism for treating obesity, it is inferred that the dietary addition of CO or MCFA can regulate energy metabolism. Therefore, to test the hypothesis, the energy metabolism level of HFD-induced C57BL/6J mice is determined, while the thermogenesis capacity and BAT activity are assessed. Furthermore, the marker genes of BAT thermogenesis are analyzed to clarify the potential mechanism behind the anti-obesity effect of CO and MCFA.

## Materials and methods

### Animals and experimental design

Forty-eight male C57BL/6J mice (4–6 weeks old, weighing 20–22 g) were purchased from Beijing Vital River Laboratory Animal Technology Co., Ltd (China). The mice were housed four per cage during this experiment and were maintained in a 12 h light/dark cycle in standard laboratory conditions at an ambient temperature of 22°C ± 1°C and relative humidity of 60% ± 10%. The animals were acclimatized for a week and provided a standard laboratory chow diet with *ad libitum* access to water. They were then randomly divided into four groups of 12 mice according to body weight, following an adaptation period. Each group was assigned one of four dietary treatments for 17 weeks as follows: The LFD group (low-fat diet, 10% of the calories were derived from fat), the HFD group (100% HFD, 60% of the calories were derived from fat), the HFD + CO group (95% HFD + 5% CO, 5% CO instead of lard was mixed with the HFD, w/w), and the MCFA group (97.5% HFD + 2.5% MCFA, 2.5% MCFA instead of lard was mixed with the HFD, w/w). Briefly, the HFD (Research Diets Inc. #D12492), which provided 20% protein, 20% carbohydrates, and 60% fat in terms of calories, was modified by adding purified CO or MCFA. Then, either 5 or 2.5% of lard, which was used as a fat source in the regular HFD, was replaced with either 5 or 2.5% CO or MCFA (Table 1). The mice had *ad libitum* access to food and drinking water. Body weight gain and food intake were assessed once a week.

**TABLE 1:** Ingredient composition of the diets fed to mice.

	LFD		HFD		HFD + CO		HFD + MCFA	
	g	kcal	g	kcal	g	kcal	g	kcal
Casein	200	800	200	800	200	800	200	800
L-Cystine	3	12	3	12	3	12	3	12
Corn starch	315	1,260	0	0	0	0	0	0
Maltodextrin	35	140	125	500	125	500	125	500
Sucrose	350	1,400	68.8	275.2	68.8	275.2	68.8	275.2
Cellulose	50	200	50	200	50	200	50	200
Soybean oil	25	225	25	225	25	225	25	225
Lard	20	180	245	2,205	232.5	2092.5	238.875	2149.875
Mineral mix	10	0	10	0	10	0	10	0
Dicalcium phosphate	13	0	13	0	13	0	13	0
Calcium carbonate	5.5	0	5.5	0	5.5	0	5.5	0
Potassium citrate	16.5	0	16.5	0	16.5	0	16.5	0
Vitamin mix	10	40	10	40	10	40	10	40
Choline bitartrate	2	0	2	0	2	0	2	0
FD&C yellow dye	0.05	0	0.05	0	0.05	0	0.05	0
CO	0	0	0	0	12.5	112.5	0	0
MCFA	0	0	0	0	0	0	6.125	55.125
Total	1055.05	4257	773.85	4257.2	773.85	4257.2	773.85	4257.2

The animals were treated humanely according to the guidelines of the Animal Ethics Committee of China Agricultural University, which approved the study (Ethics reference number: AW20059102-4). The principles of laboratory animal care were followed, and all procedures were conducted according to the guidelines established by the National Institute of Health, while every effort was made to minimize suffering.

At the end of the experimental process, mice fasted for 16 h were sacrificed via cervical dislocation. The blood was collected in tubes with ethylene diamine tetra acetic acid (EDTA) (5 mM final concentration) and protease inhibitors, after which it was centrifuged at 3,000 rpm for 15 min at 4^°^C for plasma collection. The biochemical plasma parameters, including triglycerides (TG), total cholesterol (TC), low-density lipoprotein cholesterol (LDL-C), and high-density lipoprotein cholesterol (HDL-C) levels, were measured using a Hitachi automatic analyzer (Hitachi, Ltd., Tokyo, Japan) with 100 μL heart blood plasma. The liver, epididymal (EP) white adipose tissue (WAT), subcutaneous (SUB) WAT, and interscapular BAT of each mouse were carefully dissected and weighed. The tissues isolated for gene expression analyses were collected rapidly, frozen with liquid nitrogen, and kept at –80°C. The tissues isolated for histology and immunohistochemistry experiments were immediately fixed in 4% paraformaldehyde (PFA) and embedded in paraffin for sectioning.

### The gas chromatography analysis of the coconut oil fatty acid composition and the medium-chain fatty acids formulation

The CO was donated by the Hainan Baoting Yezefang Food Co., Ltd. (Hainan, China). The fatty acid composition and corresponding quantity content of the sample CO were tested using gas chromatography (GC) according to the Chinese National Standard GB/T 17377-2008. Previously established proportions and results involving the fatty acid components of CO were followed to formulate the sample MCFA, which consisted of octanoic acid and decanoic acid (purity ≥ 99.5%; Sigma, China), at a ratio of 3:1, as previously described (22).

### The metabolic rate, physical activity, and respiratory gas analysis

The oxygen consumption and physical activity of the mice were determined after a 16-week treatment before performing the glucose tolerance test (GTT) (23). The oxygen consumption measurements were conducted using the TSE Lab Master System (24). All mice were acclimatized for 24 h before performing the measurements. Each mouse was placed in a metabolic chamber (210 cm^2^, 11.5 cm in height), after which the O_2_ consumption (VO_2_, mL/kg/h) and CO_2_ production (VCO_2_, mL/kg/h) volumes were continuously recorded over the next 24 h. The air from each chamber was sampled for 1 min every 11 min, and the data were stored in a spreadsheet. The mice were maintained at 25^°^C in a 12 h light/dark cycle with free access to food and water. Their physical activity was measured for 24 h using the optical beam technique (Opto-M3; Columbus Instruments, Columbus, OH, USA) and calculated as a 24 h average activity.

The respiratory exchange ratio (RER) and Energy expenditure (EE) was then calculated according to the following formula, provided by the manufacturer and previous studies (25, 26):


RER=VCO/2VO.2

EE(kcal)=[3.815VO(L/min)2+1.232VCO(L/min)2]1440min.

### The intraperitoneal glucose tolerance test and insulin tolerance test

The GTT and insulin tolerance test (ITT) were conducted as described previously (27). After a 16-week treatment, a GTT was performed on mice fasted for 16 h with free access to water. A Roche Diabetes Care glucometer (Roche, Germany) was employed to measure the glucose concentrations at 0, 15, 30, 60, 90, and 120 min after a 2.5 U/kg ● body weight glucose injection, using blood collected via venous bleeding from the tail vein.

After a 17-week treatment, an ITT was performed on mice fasted for 6 h with free access to water. The glucose concentrations were measured at 0, 15, 30, 60, 90, and 120 min after insulin injection (0.8 U/kg ● body weight, Novolin, USA), using blood collected via venous bleeding.

### Cold-induced thermogenesis and rectal temperature measurement

The body temperature of each mouse was measured at room temperature during the 17-week treatment using a rectal probe connected to a digital thermometer (Yellow Spring Instruments, China). The cold tolerance tests were performed by placing the mice in a cold chamber (4°C) with free access to food and water, as described in previous studies (28, 29). The rectal temperature was then measured at 1, 2, 3, and 4 h, respectively, after exposure to the chamber.

### Positron emission tomography-computed tomography

At the end of the experiment, the mice were left unfed overnight. After exposure to a cold environment (4°C) for 30 min, the mice were lightly anesthetized with isoflurane and injected with ^18^F-fluorodeoxyglucose (F-FDG) (500 mCi) via the tail vein. They were then subjected to positron emission tomography-computed tomography (PET-CT) imaging 60 min after the radiotracer injection, using the Siemens Inveon Dedicated PET System and the Inveon Multimodality System (CT/SPECT) (Siemens Preclinical Solutions, Knoxville, TN, USA) at the Institute of Laboratory Animal Sciences, Chinese Academy of Medical Sciences. The Inveon Acquisition Workplace software was used for the scanning process. The PET-CT instrument parameters and data analysis adhered to previously described methods (24).

### Histological analysis and immunohistochemical staining

The tissues fixed in 4% PFA were sectioned into 7 μm after being embedded in paraffin. Multiple sections were prepared and stained with hematoxylin and eosin (H&E) for general morphological observation (five slides per mouse and six mice per group). One section was taken from each mouse for cell diameter measurement and five different fields of the section were selected randomly and photographed; the photographs were analyzed in Image-J.

The tissue sections for immunohistochemical testing were prepared on pretreated poly-L-lysine coverslips. Immunohistochemical staining was performed according to the standard protocol using UCP1 at a 1:100 dilution. The samples were incubated overnight in a humidified chamber at 4°C. The secondary antibodies for immunohistochemical staining were purchased from Invitrogen. All images were acquired using an Olympus BX51 system and processed in Image-J.

### RNA extraction and quantitative real-time polymerase chain reaction

The total RNA was extracted using TRIzol™ Reagent (Invitrogen, USA) according to the instructions of the manufacturer. As described in previous studies (29), the isolated RNA was quantified by measuring the OD at 260 nm and 280 nm using a microplate reader (Multiskan GO, USA). The reverse transcription of the total RNA (2.5 μg) was performed using a high-capacity cDNA reverse transcription kit (Takara Biotechnology Co., Ltd., China). Quantitative real-time polymerase chain reaction (qPCR) was conducted in triplicate for each sample and analyzed using a LightCycler 480 real-time PCR system (Roche, Germany) with SYBR Premix Ex Taq™ II (Takara Biotechnology Co., Ltd., China). Calculated the relative gene expression using the 2^–ΔΔCT^ method.

### Statistical analysis

All the data reported in this paper was expressed as the means ± SEM. The analysis was performed via one-way ANOVA, followed by a *post hoc* Tukey’s test or Duncan’s test, using SPSS 27.0. A *p*-value less than 0.05 was considered statistically significant.

## Results

### Medium-chain fatty acids were the main component in the coconut oil sample

The fatty acid profile in the CO sample was analyzed using chromatography to explore the potential primary active ingredient (10). The contents (%) of the CO components are shown in Table 2. Lauric acid (49.4%) was the most abundant fatty acid and myristic acid (17.7%) was the second dominant fatty acid in the CO sample. Octanoic acid (8.4%), palmitic acid (8.2%), decanoic acid (6.7%), oleic acid (5.3%), stearic acid (3.2%), and linoleic acid (0.9%) were also identified. Octanoic acid (C8:0) and decanoic acid (C10:0) were representative MCFA components in the CO sample due to the classification of lauric acid was controversial as mentioned before. Therefore, the MCFA sample were formulated with octanoic and decanoic acid at a ratio of 3:1 according to proportions provided by previous studies (22). The ability of CO to restrict obesity and whether its main component, MCFA, played a significant role were explored.

**TABLE 2:** The quantity contents (%) of the component in sample CO and MCFA.

Components	CO content (%)	MCFA content (%)
Octanoic acid (C8:0)	8.4	75
Decanoic acid (C10:0)	6.7	25
Lauric acid (C12:0)	49.4	/
Myristic acid (C14:0)	17.7	/
Palmitic acid (C16:0)	8.2	/
Palmitoleic acid (C16:1)	<0.05	/
Heptadecanoic acid (C17:0)	<0.05	/
Heptadecenoic acid (C17:1)	<0.05	/
Stearic acid (C18:0)	3.2	/
Oleic acid (C18:1)	5.3	/
Linoleic acid (C18:2)	0.9	/

### Coconut oil and medium-chain fatty acids reduce high fat diet-induced body weight gain

**FIGURE 1:**
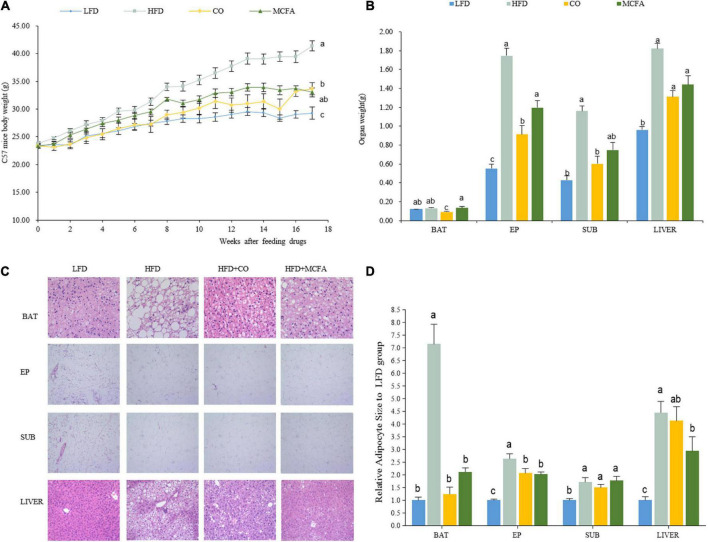
CO and MCFA reduce body weight gain. **(A)** Weekly body weight (*n* = 12). **(B)** The organ weights of the LFD, HFD, CO + HFD, and MCFA + HFD mice (*n* = 12). **(C)** HE staining of BAT (×40), EP WAT (×10), SUB WAT (×10), and liver (×20) of the LFD mice, HFD mice, and HFD mice treated with either CO or MCFA. **(D)** Relative adipocyte size to LFD group. Values represent means ± SEM. Mean values with different letters are significantly different (*P* < 0.05).

CO displays many proven benefits. This study aimed to verify whether CO or its main ingredient, MCFA, could inhibit HFD-induced obesity and metabolic syndrome. C57BL/6J mice were fed an HFD with and without 5% CO or 2.5% MCFA to evaluate and compare their anti-obesity effect. At the beginning of the experiment, the body weights of the four groups were similar. However, the final body weight of the HFD group was significantly higher than in the CO and MCFA groups. CO significantly reduced HFD-induced weight gain from the seventh week of the study until the end, while its significance was not evident in the MCFA group until week 11. The MCFA group exhibited a slightly lower weight than the CO group at week 17, which was 33.02 ± 1.66 g and 33.68 ± 2.24 g, respectively (Figure 1A). Furthermore, a significant decline in the fat mass of the CO and MCFA animals was also noticeable. Compared with the LFD group, the average SUB WAT, EP WAT, and liver weight of the HFD group increased the most. CO was responsible for a more significant attenuation (Figures 1B,C). The decline in body weight gain seemed primarily due to a reduced SUB and EP WAT mass. CO was considerably more effective in reducing the WAT. The histological analysis also showed that the lipid droplet sizes in the BAT of the CO and MCFA groups were smaller than in the HFD mice, although the MCFA group was weaker than the CO group (Figure 1D). MCFA significantly alleviated lipid deposition in the liver (Figures 1C,D). These results suggested that CO and MCFA could effectively obstruct weight gain while affecting the adipose tissue composition of HFD-induced obese mice.

### Coconut oil and medium-chain fatty acids improve insulin sensitivity

**FIGURE 2:**
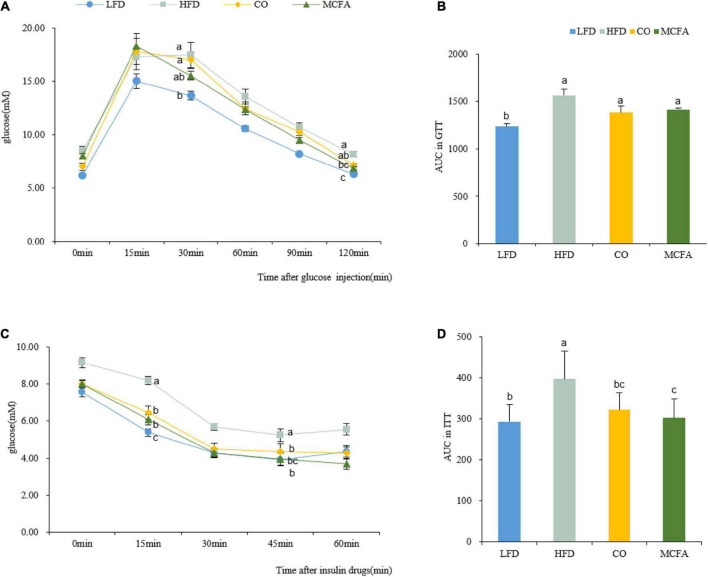
CO and MCFA ameliorate glucose tolerance and insulin sensitivity. **(A)** GTT performed on the LFD, HFD, CO + HFD, and MCFA + HFD mice (injected with 2.5 U glucose per kg after a 16 h fast) (*n* = 8–12). **(B)** Average area under the curve (AUC) (*n* = 8–12). **(C)** The ITT was evaluated based on injection with 0.8 U insulin per kg after 6 h of fasting (*n* = 8–12). **(D)** Average area under the curve (AUC) (*n* = 8–12). Values represent means ± SEM. Mean values with different letters are significantly different (*P* < 0.05).

Obesity always involves deviant glucose homeostasis and biochemical plasma parameters. GTT and ITT were conducted to assess whether CO and MCFA could normalize glucose metabolism. MCFA and CO treatment effectively improved insulin sensitivity as determined by the area under the curve (AUC) analysis, while the glucose metabolism exhibited no noticeable improvement (Figure 2). The MCFA and CO treatment showed similar results in limiting the HFD-induced hyperinsulinemia (Figures 2C,D). The plasma biochemistry, including the TC, TG, HDL-C, and LDL-C levels, were increased substantially in the HFD group (Table 3). CO and MCFA treatment reduced the HDL-C and LDL-C which were enhanced by HFD, although the TC in the MCFA group remained unaffected. These results suggested that CO and MCFA increased insulin sensitivity, and partially ameliorated the biochemical plasma parameters in the HFD-induced obese mice.

**TABLE 3:** Plasma lipid profile of mice.

	TC (mM)	TG (mM)	HDL-C (mM)	LDL-C (mM)
LFD	2.58 ± 0.90^b^	0.34 ± 0.02^b^	2.09 ± 0.08^c^	0.24 ± 0.01^d^
HFD	4.33 ± 0.20^a^	0.64 ± 0.11^a^	2.93 ± 0.22^a^	1.38 ± 0.12^a^
CO + HFD	3.10 ± 0.18^b^	0.17 ± 0.05^c^	2.08 ± 0.15^c^	1.00 ± 0.08^b^
MCFA + HFD	3.46 ± 0.16^ab^	0.29 ± 0.04^bc^	2.53 ± 0.13^b^	0.81 ± 0.08^c^

The plasma lipid profiles were measured after overnight fast in represent means ± SEM (n = 10–12).

Mean values with different letters are significantly different (p < 0.05).

### Coconut oil and medium-chain fatty acids increase energy expenditure

**FIGURE 3:**
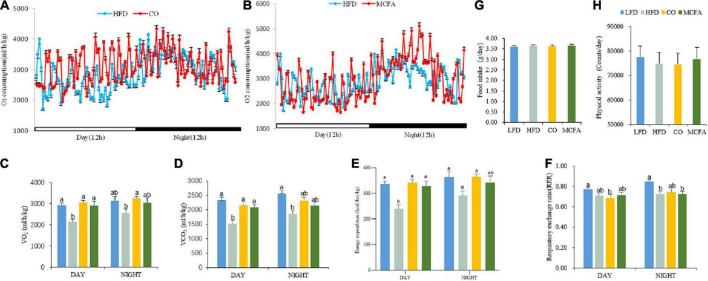
CO and MCFA increase energy expenditure in obese mice. **(A,B)** The energy expenditure of the mice treated with either CO or MCFA is examined by measuring the VO_2_ over 24 h. Bar graphs represent the average for **(C)** oxygen consumption and **(D)** carbon dioxide production, (*n* = 10). **(E)** Energy expenditure. **(F)** RER (RER = VCO_2_/VO_2_). **(G)** Food intake. **(H)** Physical activity (*n* = 10). Values represent means ± SEM. Mean values with different letters are significantly different (*P* < 0.05).

The previous section showed that CO and MCFA could inhibit HFD-induced body weight gain and improve insulin sensitivity. Energy balance fluctuations contribute to the amelioration of obesity (19). Therefore, the food intake and physical activity of the mice treated with CO or MCFA were quantified to assess the exact cause of converted body weight gain, and there were no significant differences between any of the groups (Figures 3G,H). Next, possible changes in the metabolic energy rate were verified using a comprehensive lab animal monitoring system. The mice in the CO group showed markedly higher VO_2_ and VCO_2_ levels during the 12 h light/dark cycle than the HFD mice (Figures 3A–D), while the MCFA group showed weak enhancement. In addition, the CO group exhibited a significant increase in EE both during the day and at night while the MCFA group showed a kindly increase in energy metabolism at night (Figure 3E). These results indicated that the mice in the CO and MCFA consumed more energy than the HFD mice, it is worth noting that the consumption of the CO group was close to that of the LFD mice. However, the CO and MCFA groups showed no significant increases in the RER compared with the HFD group (Figure 3F), suggesting the energy source is still mainly fat. These results showed that CO and MCFA reversed HFD-induced weight gain by elevating energy consumption instead of influencing food intake and physical activity.

### Coconut oil promotes thermogenesis more than medium-chain fatty acids

**FIGURE 4:**
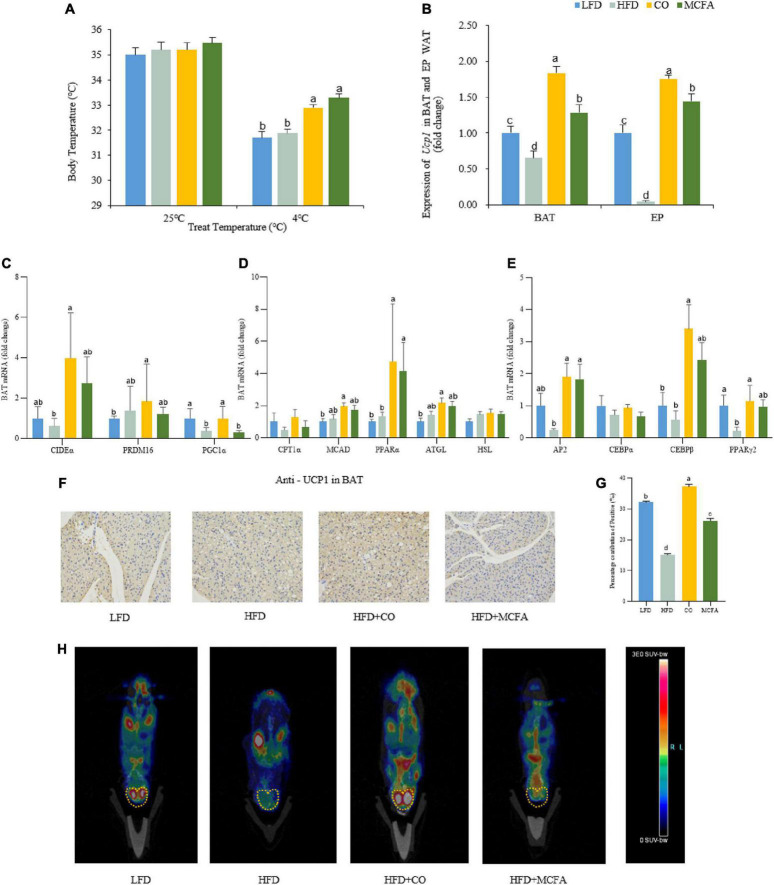
CO and MCFA maintain core body temperature by increasing BAT activity. **(A)** The core body temperature of the treated mice at room temperature (25°C) and cold challenge conditions (4°C for 4 h) (*n* = 12). **(B)** Ucp1 expression profile in BAT and EP WAT, real-time PCR analysis of Ucp1 expression (*n* = 12). **(C–E)** Gene expression profile in BAT, real-time PCR analysis of thermogenic, lipolytic, fatty oxidation, fatty acid synthesis and browning-related gene expression profile. **(F,G)** Immunohistochemistry for the UCP1 protein (brown stain) in the BAT sections of CO and MCFA-treated mice (*n* = 10). Original magnification: × 200. **(H)** PET-CT image after injection of ^18^F-FDG into the LFD mice, the HFD mice, and HFD mice treated with either CO or MCFA for 16 weeks. The yellow dotted line indicates the anatomical site of the interscapular BAT (*n* = 5). Values represent means ± SEM. Mean values with different letters are significantly different (*P* < 0.05).

Energy can be expended by performing work or via thermogenesis (30). Given that no difference in physical activity was observed, a cold tolerance test was conducted to investigate the capacity of adaptive thermogenesis to further evaluate the EE among the four groups of mice. At room temperature, the body temperatures of the four groups determined via rectal temperature measurements showed no differences. However, exposure to a cold environment (4°C) for 4 h caused a considerable decline in the body temperature of the HFD group compared to the CO and MCFA groups (Figure 4A). These results suggested that CO and MCFA treatment can increase the thermogenic capacity of mice, which may provide a plausible explanation for their increased EE.

WAT typically stores energy while BAT consumes energy (31). BAT can convert energy into heat, which is essential for non-shivering thermogenesis (32). Therefore, it was hypothesized that CO or MCFA might activate the BAT in the HFD-induced obese mice, and the expressions of molecules associated with thermogenesis and lipid metabolism in the BAT and browning in the EP and SUB WAT were examined. Firstly, it was found that CO and MCFA treatment indeed upregulated the expression of the thermogenesis-key gene UCP1, while it was down-regulated in the BAT of the HFD group. Since WAT browning played an essential role in inducing EE, the levels of the browning biomarker, UCP1, were also analyzed in the EP and SUB WAT. The results showed that CO and MCFA elevated the expression of UCP1 in EP WAT only (Figure 4B). Furthermore, CIDEα, PRDM16, and PGC1α (thermogenesis-related genes) were all up-regulated in the BAT of the CO group (Figure 4C). In addition, fatty acids are the primary sources of fuel for the thermogenesis of BAT. CO administration more effectively accelerated the oxidation of fatty acids analyzed by the gene expression of MCAD, PPARα, and ATGL (Figure 4D). CO can also significantly up-regulate the expression of AP2, C/EBPβ, and PPARγ2 while it has no significant effect on other brown adipogenesis-related genes such as C/EBPα (Figure 4E).

This study also examined whether CO or MCFA upregulated the UCP1 protein expression in BAT. The immunohistochemistry results indicated that the positive staining of UCP1 declined in the HFD group, while the CO group was significantly stained after treatment, and the MCFA was relatively weak (Figures 4F,G). The UCP1 protein level in the BAT of the CO group was enhanced compared with the HFD group. The BAT activity in the mice could be observed using ^18^F-FDG and micro PET-CT (30). The PET-CT images indicated that the CO group exhibited the highest standard uptake values (SUV) at the interscapular position of the mice (Figure 4H). Taken together, these results demonstrate that CO can activate BAT heat production capacity and enhance the fatty acid oxidation function of BAT, thereby increasing the overall energy consumption of the mice and achieving the purpose of weight control.

## Discussion

This study characterized the impact of CO and MCFA on the metabolic health of mice. It examined whether using CO or MCFA to replace lard in an HFD can restrict obesity and subsequent metabolic syndrome. Moreover, the results indicated that CO application reduced body weight gain by decreasing the weight of adipose tissue and adipocyte size, elevating EE and thermogenesis by enhancing BAT activity, and combating related metabolic diseases. Furthermore, replacing lard with MCFA had the same metabolic benefit, but attenuated the activation effect on BAT compared to CO.

Obesity is a risk factor that threatens human health worldwide. In modern society, obesity is caused by an imbalance between energy intake and expenditure. Common medications and surgery for treating obesity are often accompanied by side effects or are difficult to adhere to. Increasing thermogenesis to enhance energy metabolism is a novel way to inhibit obesity by switching energy to heat rather than triacylglycerols. Furthermore, activating BAT is a potential therapeutic strategy for ameliorating obesity. CO is a type of saturated fat that has been significantly promoted as a healthy oil. Consistent with previous research (10, 33), the CO used in this study was rich in lauric acid (C12:0) and myristic acid (C14:0), accounting for 49.4 and 17.7% of the total fatty acids, respectively (Table 2). As mentioned earlier, the classification of lauric acid(C12:0) is still controversial. The MCFA in this study are composed of its typical representatives, octanoic acid (C8: 0) and decanoic acid (C10:0), which accounted for 8.4 and 6.7% of the total fatty acid content in CO, respectively (33). This study sought to examine the effect of CO and its main component MCFA on obesity. The MCFA consisted of octanoic acid and decanoic acid at a 3:1 ratio and was prepared according to previous research and the CO composition (22). A 5% CO or 2.5% MCFA value was selected to replace the lard in the HFD according to previous studies showing that one-third to one-half of fatty acids such as the MCFA in CO were absorbed, and the 2015–2020 Dietary Guidelines for Americans (DGA) recommended to limit saturated fats to less than 10% of total calories daily (34–37). Moreover, further animal and human trials must determine the appropriate dose for CO and MCFA intake.

This study evaluated the inhibitory effect of CO and MCFA on obesity. The data demonstrated that CO and MCFA reduced body weight gain, especially the size and weight of EP and SUB WAT while decreasing lipid deposition in the liver (Figure 1). Therefore, supplementation with CO and MCFA displayed the potential to improve fatty liver disease (11). Similar results were also observed in sexually mature female mini-pigs fed a high-fat, high-fructose diet replaced with 5% CO or lard. The CO group showed less evidence of fatty liver disease and less deep belly fat than the lard group (38). These results were verified by a healthy human-based survey, indicating that the consumption of CO as part of a balanced diet resulted in increased fat-free mass (10). The MCFA-induced reduction of fat mass in C57BL/6J mice fed an HFD was confirmed in a recent study (22). A previous report indicated that CO treatment exacerbated hepatic lipid accumulation (33). This disparity could be attributed to the animal model, dosing method, or length of drug administration. In this study, the C57BL/6J mice received CO as a dietary supplement for 17 weeks. Conversely, in a previous, seemingly contradictory study, Wistar rats were supplemented with CO by gavage for 30 d after 12 weeks of receiving an HFD. These results also suggested that the best way to use CO supplementation for lipid accumulation reduction was via the daily diet since applying CO might even be counterproductive if obesity had already developed.

Furthermore, CO and MCFA ameliorate insulin sensitivity. Investigations involving humans confirmed that CO treatment could increase insulin sensitivity (10). The inclusion of MCFA in an obesogenic diet can also prevent hepatic lipid accumulation and lower insulin resistance indices (11). Similarly, CO and MCFA supplementation can improve insulin resistance (Figure 2). Furthermore, the present study showed that HFD altered the plasma profiles, such as increasing the TC, HDL-C, and LDL-C levels, and that these anomalies were reversed by CO and MCFA (Table 3), which consistent with previous studies have confirmed that CO can reduce TC, LDL-C, and HDL-C levels in plasma (7, 39). The role of CO in human nutrition is still controversial since CO can increase HDL-C compared with plant oils such as olive oil (40). However, CO demonstrated a better lipid profile compared with animal oils, such as lard used in this study (7). The differential effects of CO on cardiovascular health can be attributed to the diversity of the compared objects, necessitating more research that focuses on diverse populations and objects to normalize and establish a unified measurement standard. In general, these results demonstrate that CO and MCFA supplementation can provide a dietary approach for attenuating HFD-induced obesity and its metabolic alterations.

CO and MCFA are essential in treating metabolic syndromes, although their exact mechanism remain elusive. Considering that obesity is caused by an energy imbalance, the food intake and physical activity of the mice were examined, showing no significant differences (Figures 3G,H). Furthermore, the CO and MCFA groups exhibited enhanced EE compared to the HFD group (Figures 3A–E). BAT can burn energy into heat, while energy consumption is essential for maintaining body temperature (19). Furthermore, BAT activation promoted weight loss (41). Mice treated with HFD, CO, and MCFA at room temperature maintained high core body temperatures compared to the LFD group, although no marked differences were evident (Figure 4A). But when a cold tolerance test was performed on the mice to characterize the BAT activity *in vivo*, CO and MCFA treatment can significantly increase the core body temperature when the animals were subjected to cold stimuli (4^°^C, 4 h) compared to the HFD group (Figure 4A). The findings suggest that CO and MCFA treatment regulate thermogenesis in the mice, helping to maintain their body temperature by expending excess energy, thus counteracting obesity.

However, further exploration of the mechanisms involved is required. BAT thermogenesis relied on UCP1, which activity in BAT is reduced due to the development of obesity (30). Consistent with this, UCP1 mRNA expression in the HFD group showed a downward trend compared with the LFD group (Figure 4B). Controversially, several studies have also identified that HFD feeding increases UCP1 mRNA expression in BAT (42). Possible factors for these inconsistencies include mouse strain, ambient temperature, and even dietary composition. It has been noted that when comparing different studies using the same mouse strain and temperature, the results are not reproducible and vary in seemingly arbitrary ways (43). CO and MCFA treatment not only reversed the decline in UCP1 mRNA expression in BAT, but also reversed UCP1 expression in EP WAT (Figure 4B), indicating that CO and MCFA could activate BAT and drive the browning of EP WAT in HFD-induced obese mice. In this study, we also found that the expression of thermogenesis EE -related genes in the BAT of mice such as CIDEα, PRDM16, and PGC1α, as well as genes associated with fatty acid oxidation (FAO) including CPT1α, MCAD, PPARα, ATGL, and HSL, can be upregulated by CO (Figure 4D). CO can also significantly up-regulate the expression of brown adipogenesis-related genes such as AP2 and C/EBPβ (Figure 4E). These findings suggest that CO might be more effective in increasing BAT activity, rather than MCFA treatment in this study. Complex components of CO and its possible synergistic effects may be the key to increasing BAT activity.

CO also upregulated the protein level of UCP1 in BAT, which was consistent with its transcriptional levels (Figures 4F,G). The PET-CT image showed that the PET signal was stronger in the interscapular position of the CO-treated mice (Figure 4D). These results demonstrate that CO can increase the activity of BAT in HFD-induced obese mice. Therefore, these results support the hypothesis that CO restricts obesity while the underlying mechanism involves increased EE by increasing BAT activity, while the activation of MCFA on BAT produces a little effect. The previous study has shown that medium-chain triglycerides (MCT), the binding state of MCFA, could also activate hepatic BAT thermogenesis and reduce metabolic disorders (15). Under the research conditions of this experiment, MCFA drastically deplete hepatic lipid droplets consistent with previous findings (Figure 1C). MCFA had similar BAT weight, histology, and UCP1 mRNA expression with the CO group, but not protein and PET-CT. MCFA may affect the modification of UCP1 protein during translation, resulting in its inability to exert its thermogenesis normally. Whether the state of MCFA affects its activation of BAT is also worth exploring. MCFA may also play a role in maintaining body temperature and increasing energy consumption by inducing WAT browning and triggering liver thermogenesis, thereby improving the metabolic features associated with obesity.

The findings indicate that the amelioration of obesity by CO is only partially attributable to MCFA. The conclusion regarding the relationship between pure MCFA and obesity cannot be directly applied to CO due to its complex ingredients, even though many studies have employed CO as experimental material to infer the role of MCFA. It is surmised that other substances in CO, in addition to the MCFA [consists of octanoic acid (C8:0) and decanoic acid (C10:0)], also contribute to combating obesity, such as lauric acid (C12:0), which accounts for 49.4% of CO. Recent research demonstrated that lauric acid supplementation reduced hepatic steatosis and insulin resistance despite increased plasma profiles (44). Moreover, impaired body energy metabolism and weakened BAT thermogenesis of HFD-fed mice were improved by lauric acid (C12:0) supplementation (45). On the one hand, the difference between MCFA and CO could be explained by lauric acid (C12:0) properties; on the other hand, MCFA may have a synergistic effect with lauric acid(C12:0). CO also contains myristic acid, palmitic acid, and oleic acid, which have received minimal attention. The limitation of this experiment is that there is no further verification on whether MCFA and other substances in CO play a synergistic role in anti-obesity.

This study demonstrated that two dietary fats (lard and CO), mainly containing saturated fat, seemingly displayed different health effects. This may be attributed to a difference in fatty acid profiles. CO comprises 49% lauric acid and 17.7% myristic acid, while lard consists of 38.24% oleic acid and 22.68% palmitic acid (8). The influence of dietary fats on metabolic indicators may vary according to their general classification of saturated or unsaturated fatty acids, as well as their primary constituent fatty acids, processing methods, and how they are consumed.

Certain questions remain significant for future studies. First, only male mice were used in this study. Whether CO and MCFA are beneficial for females as well should be investigated, given that sexual dimorphism has previously been observed in relation to dietary restrictions (46). Second, future research should focus on screening the downstream metabolites of CO and examine their anti-obesity effects when employed in a singular capacity or conjunction with MCFA. Furthermore, this study focused only on the important thermogenesis marker expression profile in BAT, rather than WAT. More importantly, a growing body of research has shown that diet alters crosstalk between the gut microbiota and host metabolism (47–49). Therefore, the mechanism of regulating obesity and related syndromes by modifying gut microorganisms with CO is worth further development.

## Conclusion

Over the past decade, the consumption of CO and its main ingredient, MCFA, have increased rapidly, partly because these products provide health benefits. Many previous studies have paid more attention to the effect of CO on cardiovascular risk. However, this study proves that CO exhibits a distinct anti-obesity effect while revealing the mechanism as BAT activation and increased EE in an HFD-induced obese mice model. Furthermore, it is worth pointing out that CO has a better effect than that MCFA composed of octanoic acid and decanoic acid. MCFA may cooperate with other substances in CO to increase the anti-obesity effect, such as lauric acid. As discussed above, this work provides evidence for the health benefits of CO and MCFA and is essential for developing safe therapeutic approaches to combat obesity and related lesions. CO, rich in MCFA and other beneficial ingredients, is a better alternative to lard.

## Data availability statement

The raw data supporting the conclusions of this article will be made available by the authors, without undue reservation.

## Ethics statement

This animal study was reviewed and approved by the Animal Ethics Committee of China Agricultural University.

## Author contributions

YG and YY contributed to collecting and analyzing the data and writing the manuscript. YL, JG, and XH contributed to executing experiments and collecting data. FZ contributed to collecting data. YY, WH, and JZ contributed to designing the research and provided essential materials. YY contributed to reviewing the manuscript. All authors contributed to the article and approved the submitted version.

## Abbreviations

AT: adipose tissue
ATP: adenosine triphosphate
BAT: brown adipose tissue
CO: coconut oil
EDTA: ethylene diamine tetra acetic acid
EP WAT: epididymal white adipose tissue
F-FDG: F-fluorodeoxyglucose
GC: gas chromatography
GTT: glucose tolerance test
HFD: high fat diet
ITT: insulin tolerance test
LCFA: long-chain fatty acids
LFD: low fat diet
MCFA: medium-chain fatty acids
qPCR: quantitative real-time polymerase chain reaction
RER: respiratory exchange ratio
SUB WAT: subcutaneous white adipose tissue
UCP1: uncoupling protein 1
VCO2: volume of CO2 consumption
VO2: volume of O2 consumption
WAT: white adipose tissue
β 3-AR: β 3-adrenergicreceptor.
