# Supplementary material for: Coconut oil and medium-chain fatty acids attenuate high-fat diet-induced obesity in mice through increased thermogenesis by activating brown adipose tissue
Source: Front Nutr. 2022 Oct 28;9:896021. doi: 10.3389/fnut.2022.896021 (PMC9650104; doi:10.3389/fnut.2022.896021)
Supplement: Supplementary file 1 [file Table_1.DOCX]

Supplementary Material

# Supplementary Tables

**Table S1.** The sequences of oligonucleotide primers.

| **Genes** | **Primer Sequence (5’→3’)** |
| --- | --- |
| *Ucp1* forward | AGGCTTCCAGTACCATTAGGT |
| *Ucp1* reverse | CTGAGTGAGGCAAAGCTGATTT |
| *β-actin* forward | CGTTGACATCCGTAAAGACC |
| *β-actin* reverse | AACAGTCCGCCTAGAAGCAC |
